# The C5 Variant of the Butyrylcholinesterase Tetramer Includes a Noncovalently Bound 60 kDa Lamellipodin Fragment

**DOI:** 10.3390/molecules22071083

**Published:** 2017-06-29

**Authors:** Lawrence M. Schopfer, Hervé Delacour, Patrick Masson, Jacqueline Leroy, Eric Krejci, Oksana Lockridge

**Affiliations:** 1 Eppley Institute, University of Nebraska Medical Center, Omaha, NE 68198-5950, USA; lmschopf@unmc.edu (L.M.S.); pmasson@unmc.edu (P.M.); 2 Hôpital d’Instruction des Armées Bégin-Département des Laboratoires, 69, Avenue de Paris, 94 163 Saint Mandé, France; herve.delacour@intradef.gouv.fr; 3 Neuropharmacology Laboratory, Kazan Federal University, 420000 Kazan, Russia; 4 CNRS Université Paris Descartes Cognition Action Group UMR 8257, 75006 Paris, France; jacma.leroy@parisdescartes.fr (J.L.); eric.krejci@univ-paris5.fr (E.K.)

**Keywords:** lamellipodin, *RAPH1*, butyrylcholinesterase, tetramer, C5 phenotype

## Abstract

Humans with the C5 genetic variant of butyrylcholinesterase (BChE) have 30–200% higher plasma BChE activity, low body weight, and shorter duration of action of the muscle relaxant succinylcholine. The C5 variant has an extra, slow-moving band of BChE activity on native polyacrylamide gel electrophoresis. This band is about 60 kDa larger than wild-type BChE. Umbilical cord BChE in 100% of newborn babies has a C5-like band. Our goal was to identify the unknown, 60 kDa protein in C5. Both wild-type and C5 BChE are under the genetic control of two independent loci, the *BCHE* gene on Chr 3q26.1 and the *RAPH1* (lamellipodin) gene on Chr 2q33. Wild-type BChE tetramers are assembled around a 3 kDa polyproline peptide from lamellipodin. Western blot of boiled C5 and cord BChE showed a positive response with an antibody to the C-terminus of lamellipodin. The C-terminal exon of lamellipodin is about 60 kDa including an N-terminal polyproline. We propose that the unknown protein in C5 and cord BChE is encoded by the last exon of the *RAPH1* gene. In 90% of the population, the 60 kDa fragment is shortened to 3 kDa during maturation to adulthood, leaving only 10% of adults with C5 BChE.

## 1. Introduction

An understanding of the structure of the C5 variant of human BChE is an extension of the groundbreaking achievement of Joel Sussman and Israel Silman in 1991, when they solved the crystal structure of acetylcholinesterase [1]. This pioneering work in collaboration with Jean Massoulié contributed to an understanding of the role of proline-rich peptides in assembly of the acetylcholinesterase tetramer [2]. It also inspired the solution of the human BChE crystal structure [3]. The current manuscript is dedicated to Joel Sussman and Israel Silman in celebration of the 25th anniversary of the publication of the crystal structure of acetylcholinesterase.

For decades it was assumed that BChE is organized as a homotetramer of catalytic subunits. In fact, the wild-type human BChE tetramer is composed of 4 identical glycoprotein subunits and a noncovalently bound polyproline-rich peptide. Each BChE subunit has 574 amino acids and 9 N-linked glycans. Forty residues at the C-terminus of each BChE subunit constitute the tetramerization domain. Polyproline-rich peptides intertwine with the tryptophans in the tetramerization domain, acting as a glue that assembles 4 subunits into the BChE tetramer. The polyproline-rich peptides in wild-type human BChE range in size from 21 to 32 residues [4,5] and derive from lamellipodin (Q70E73; *RAPH1* gene). Studies with recombinant human BChE have demonstrated that BChE monomers can assemble into tetramers by addition of either short polyproline peptides (17–50 Pro residues) or long polyproline peptides with an average mass of 30,000 Da [6], in agreement with initial observations with recombinant AChE [7,8] and confirmed in skeletal muscle AChE [9].

The C5 variant of human butyrylcholinesterase (BChE) was originally identified as an extra, slow-moving band of BChE activity in nondenaturing starch gel, polyacrylamide gel, and acid agar gel electrophoresis [10,11,12]. This extra band of BChE activity is called C5 to distinguish it from isozymes C1, C2, C3 and C4 that are routinely observed in normal human serum and plasma samples by gel electrophoresis. The C1, C3 and C4 bands are monomer, dimer, and tetramer forms of BChE, whereas C2 is a BChE monomer-albumin conjugate [13].

Family studies indicated that the C5 isozyme is genetically determined, though in some instances both parents of a C5 propositus had no detectable C5 band [10]. The C5 phenotype has a frequency of about 10% in Caucasians [10]. Individuals phenotyped as C5 have 30% higher plasma BChE activity on average, but activity can range up to 200% higher than the majority of individuals. The C5 phenotype is associated with low body weight and a shorter duration of action of the muscle relaxant succinylcholine [14,15].

Before 1990, it was thought that separate genetic loci directed the production of BChE and C5 BChE. Linkage analysis demonstrated that C5 (also called CHE2) is associated with Chr 2q33–q35 [16]. Subsequent DNA studies demonstrated that there is only one gene for the BChE protein (accession P06276) and that both C5 BChE and wild-type BChE are produced by the same *BCHE* gene on Chr 3q26.1 [17,18,19,20]. DNA sequencing ruled out the possibility that the promoter or coding sequences of BChE have point mutations that could explain the higher activity and slower mobility of C5 BChE [21].

Scott and Powers proposed that the C5 enzyme is a hybrid composed of C4 and a second protein [22]. Masson et al., demonstrated that the mass of the C5 variant is ~400 kDa or about 60 kDa greater than the mass of the 340 kDa BChE tetramer [23]. Linkage analysis of the C5 variant showed that C5 is under the control of a gene on Chr 2q33–q35 [16]. A genome wide association study showed that wild-type BChE is under the control of loci on Chr 3q26 and Chr 2q33–q35 [24]. Joint control of wild-type BChE by two genes is consistent with the presence of exogenous, polyproline-rich peptides in the BChE tetramer. These polyproline-rich peptides were identified by mass spectrometry analysis of the wild-type BChE tetramer and were shown to derive from lamellipodin [4,5]. Lamellipodin (also called Ras-associated and pleckstrin homology domains-containing protein 1) is a product of the *RAPH1* gene. *RAPH1* is located on Chr 2q33. Genotyping of single nucleotide polymorphisms in the *RAPH1* gene of C5 and wild-type BChE individuals supported the hypothesis that the *RAPH1* gene is the CHE2 locus [25], that is, the C5 locus.

These analyses raise a question. By analogy with wild-type BChE, one would expect the tetrameric BChE component of C5 to contain polyproline-rich peptides from lamellipodin protein or an unidentified protein encoded on Chr 2q33. Is the extra 60 kDa protein that is associated with C5 an extended fragment of lamellipodin, or is it another protein that has been missed in the genetic analyses?

Our goal was to identify the 60 kDa protein in the C5 genetic variant of human BChE. We included umbilical cord BChE in our study because we found that 100% of all cord blood samples have a C5-like band. BChE was purified from C5 plasma and cord plasma by affinity chromatography on hupresin, followed by binding to immobilized anti-human BChE monoclonal B2 18–5. The 60 kDa protein in the immunopurified C5 complex was identified by mass spectrometry and by immunoblotting with an antibody to lamellipodin.

## 2. Results

### 2.1. C5 Visualized on A Polyacrylamide Gel

**Figure 1:**
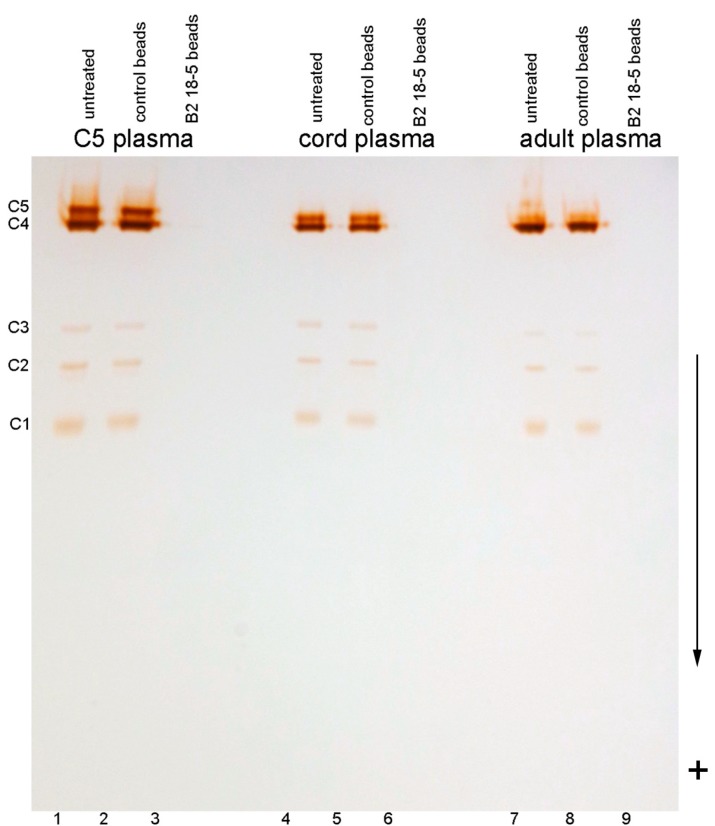
Nondenaturing gradient gel stained for BChE activity. Each lane has 3 µL plasma collected into EDTA anti-coagulant. Lanes 1, 4, 7: untreated C5 plasma, umbilical cord plasma, and adult plasma containing wild-type BChE. Lanes 2, 5, 8: plasma incubated with control Sepharose beads (no antibody). Lanes 3, 6, 9: plasma incubated with monoclonal B2 18–5 immobilized on Sepharose beads. The gel shows the location of the C5 band relative to C4, where C4 is the BChE tetramer. Umbilical cord plasma has a C5-like band that migrates slightly faster than C5. Adult plasma has a major C4 band and a shadow band above C4. The C1, C2, and C3 bands are BChE monomer, a BChE monomer-albumin conjugate [13], and dimer, respectively. Incubation of plasma with immobilized monoclonal B2 18–5 captures essentially all the BChE activity in plasma. The direction of migration is toward the positive electrode, indicated by the + sign.

BChE bands in C5 plasma of an adult are shown in Figure 1 in comparison to BChE bands from umbilical cord plasma and from a wild-type BChE adult donor. BChE activity was 4.3 u/mL in adult C5 EDTA plasma, 2.0 u/mL in cord EDTA plasma, and 2.3 u/mL in adult EDTA plasma containing wild-type BChE. All samples have bands at positions C1, C2, C3, and C4. The C5 plasma has an extra, slow moving band labeled C5 (lanes 1 and 2). Plasma from umbilical cord has an extra, slow-moving band (lanes 4 and 5) with a slightly faster mobility than the C5 band. Plasma from a normal adult (lanes 7 and 8) has a prominent C4 band and a weak shadow of activity above the C4 tetramer band. Two-dimensional electrophoresis had previously shown a strong C4 band and a slight shadow band in normal human plasma [26]. Plasma incubated with immobilized anti-human BChE monoclonal B2 18–5 has undetectable BChE activity (Figure 1, lanes 3, 6 and 9). This means that all isozymes of BChE, including C5, were captured by the antibody.

### 2.2. C5 and C5-Like Bands In Umbilical Cord Plasma

**Figure 2:**
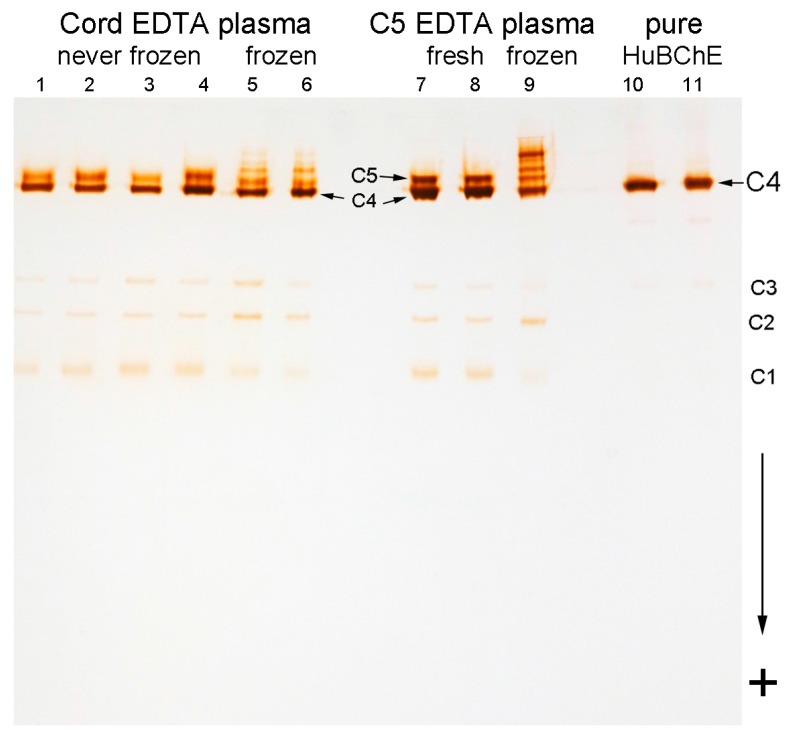
Umbilical cord plasma collected into EDTA anticoagulant has C5 and C5-like BChE. Lanes 1–4, never-frozen cord plasma; lanes 5 and 6, cord plasma stored at −20 °C for 5 years; lanes 7 and 8, never frozen C5 plasma; lane 9, C5 plasma stored at −20 °C for 9 years; lanes 10 and 11, pure human BChE from Cohn Fraction IV-4 stored at 4 °C. The native gradient gel was stained for BChE activity. Migration toward the positive electrode is indicated by the “+” sign.

Without exception, all 103 umbilical cord EDTA plasma samples had an extra band of BChE activity close to the position of the C5 band. Figure 2 shows the bands for 4 cord plasma samples (lanes 1–4). The cord plasma samples in lanes 1–3 have a C5-like band that migrates a little faster than C5. The cord plasma in lane 4 has a band at the same position as the C5 band in adult plasma (lanes 7 and 8). The frequency of the C5 band relative to the C5-like band, in cord plasma, was 1 out of 7. Cord plasma samples with a C5 band had higher BChE activity (2.2 ± 0.3 u/mL, average), compared to the activity in other cord plasma samples (1.7 ± 0.3 u/mL, average). Using 2-dimensional electrophoresis, Harris saw an extra band of BChE activity that was distinct from C5 in cord serum [26].

### 2.3. Storage Bands

Plasma stored at −20 °C for prolonged periods develops extra slow moving bands of BChE activity called storage bands. The term “storage bands” was coined by Harris [26]. Storage bands could be mistaken for C5. Cord EDTA plasma samples stored frozen at −20 °C for 5 years (Figure 2, lanes 5 and 6) and C5 EDTA plasma stored at −20 °C for 9 years (Figure 2 lane 9) had storage bands. The stored, frozen C5 sample in lane 9 is from the same donor as the fresh C5 sample in lane 8. The frozen cord plasma in lanes 5 and 6 is from different donors than the fresh cord plasma in lanes 1–4. Plasma frozen and thawed once by the Blood Bank had no storage bands (data not shown). Never-frozen C5 EDTA plasma stored at 4 °C for 2 years had faint storage bands, and retained the C5 band (data not shown).

Human BChE purified from Cohn Fraction IV-4 (Figure 2, lanes 10 and 11) consists almost exclusively of C4 tetramers. There are no storage bands, no shadow band, no C5 band, and almost undetectable C1–C3 bands. The starting Cohn Fraction IV-4 was frozen and represented pooled plasma donated by more than 500 individuals.

### 2.4. Mass Spectrometry Analysis Identifies Lamellipodin, a Proline Rich Protein Encoded by the Last Exon of the RAPH1 Gene In C5 and Cord BChE

**Figure 3:**
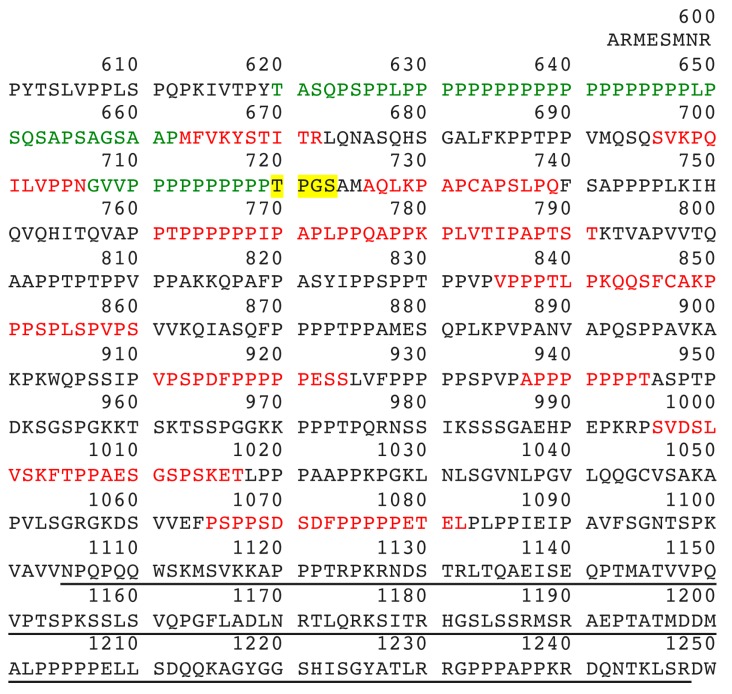
Polyproline-rich C-terminus of lamellipodin (UniProt Q70E73). Mass spectrometry analysis of BChE purified from C5 and cord plasma identified peptides that mapped to lamellipodin. Green 95–99% confidence, yellow >50% confidence, red <50% confidence. The underlined peptide, 1105–1248, was the antigen for the anti-RAPH1 antibody used for Western blotting. Wild-type BChE tetramers contain only polyproline-rich peptides encoded by residues 620–662.

Pure human BChE tetramers (C4) contain short polyproline-rich peptides encoded by residues 620–662 of lamellipodin, see Figure 3 [4,5]. Mass spectrometry has identified additional lamellipodin peptides originating from C5 and cord BChE tetramers. These peptides are encoded by the last exon of the *RAPH1* gene. They are not found in wild-type C4 tetramers. Assignment of peptides colored red in Figure 3 is tentative because the confidence of a correct assignment is less than 50%. Assignment of the green-colored peptide GVVPPPPPPPPPP (residues 707–719) is strong because confidence of a correct assignment is greater than 95%. The presence of this high-confidence peptide strengthens the assignment of lamellipodin as the extra protein in C5. However it is generally accepted that two peptides identified at 95% confidence are required for reliable assignment of a protein by mass spectrometry.

The molecular weight of the C-terminal portion of lamellipodin, i.e., that portion encoded by the last exon (ensembl number ENSE00001259426), has a mass of 68,325 Da (calculated using the MS-Digest feature of Protein Prospector). Six potential N-linked glycans could add about 13,000 Da. These observations strongly suggest that the 60 kDa unknown protein in C5 and cord BChE is a lamellipodin fragment encoded by the last exon of the *RAPH1* gene. Residues 620 to 1248 in Figure 3 may contribute to the C5 variant.

### 2.5. Capillary Electrophoresis Western Blot

**Figure 4:**
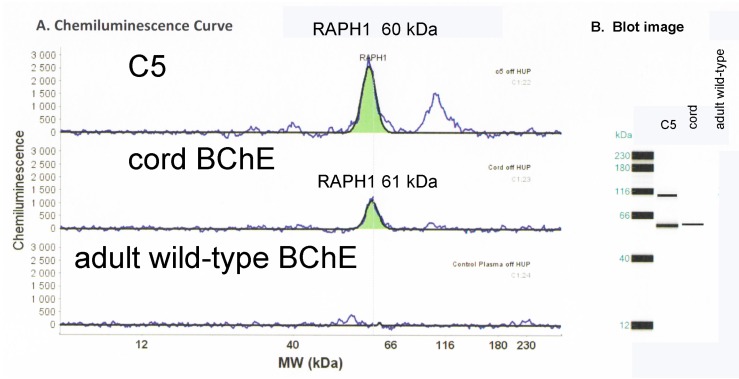
Capillary electrophoresis Western blot. Proteins in denatured samples of C5 BChE, cord BChE, and adult wild-type BChE were separated by capillary electrophoresis and hybridized with anti-RAPH1 antibody. A positive signal at 60 and 61 kDa was detected for C5 and cord BChE, respectively, but not for adult wild-type BChE. The chemiluminescent signal in panel (**A**) is depicted as a blot image in panel (**B**).

A polyclonal antibody to the C-terminus of lamellipodin recognized a 60 kDa protein from denatured C5, and a 61 kDa protein from denatured cord BChE, but did not recognize any protein from denatured adult wild-type BChE (Figure 4). The Western blot results in Figure 4 strongly support the conclusion that the extra slow moving band of BChE in C5 and cord plasma contains a nearly full length sequence encoded by the last exon of the *RAPH1* gene. A peak at around 100 kDa in Figure 4 for C5 is also recognized by the *RAPH1* antibody and may correspond to a precursor form of the proline rich protein in C5 and cord plasma.

### 2.6. Genotyping Results

Sequencing of the whole coding regions of the *BChE*, *RNPEP*, and *RAPH1* genes from two C5 BChE adults revealed no mutations in the *BCHE* and *RAPH1* genes. One patient harbored one point mutation in a compound heterozygous state in *RNPEP* (c.1735G > A, p.Val579Ile, rs3820439). This point mutation is relatively common in the population (allelic frequency of 0.33) and is considered a polymorphism. The *RNPEP* gene was sequenced because polymorphism of *RNPEP* is linked with variation in BChE activity [24]. *RNPEP* encodes arginyl aminopeptidase, (Q9H4A4) a potential protease candidate to explain the presence of a protein in C5 in place of a peptide in C4. Our results are in agreement with previous studies, which showed no mutations in the *BCHE* and *RAPH1* genes in the C5 genotype [21,25]. We conclude the C5 variant is not linked to a mutation in the *BCHE*, *RNPEP*, or *RAPH1* genes.

### 2.7. Partially Purified C5 Is Unstable

Serum samples with no anticoagulant retained the C5 band [26]. Use of heparin as an anticoagulant caused total disappearance of C5 [27]. Interaction of C5 with heparin was exploited in early affinity chromatography purifications of C5 [23]. In our laboratory, C5 plasma collected into EDTA anticoagulant and stored at 4 °C retained the C5 band for at least 2 years. A 1 mL aliquot of C5 EDTA plasma partially purified on hupresin and eluted with 0.1 M procaine in 0.1 M TrisCl pH 7.5 retained the C5 band for 2 months at 4 °C while the pH dropped to 4.5 due to hydrolysis of procaine by BChE. However, the C5 band was lost after the pH was adjusted to 7.5 with TrisCl. Our attempts to purify C5 from 330 mL of plasma resulted in disappearance of the C5 band when BChE was eluted off hupresin with 0.5 M tetramethylammonium bromide in 0.1 M TrisCl pH 7.5. Instability of the C5 band has been observed by others. Isolated C5 eluted from an agar gel migrated with C4 mobility in a second agar gel [15].

Proteolysis converts C5 to C4. Mass spectrometry analysis identified the major contaminant in hupresin-purified C5 plasma as plasminogen, at 100 kDa (data not shown). Plasminogen is the precursor of plasmin, a serine protease. Plasminogen produces small amounts of plasmin during storage [28]. Trypsin and proteases converted C5 into C4 [23]. C5 plasma treated with trypsin lost the C5 band [12]. We propose that the instability of C5 is explained by proteolytic cleavage of the long lamellipodin fragment that extends out of the protective envelope of the BChE tetramerization domain.

## 3. Discussion

### 3.1. Model of C5 BChE

**Figure 5:**
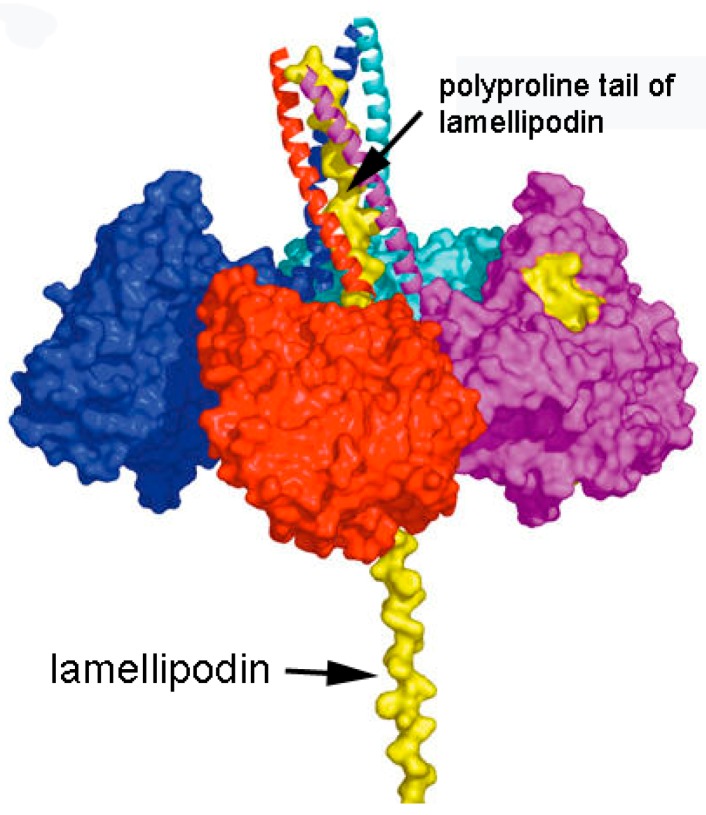
Model of C5 BChE tetramer. The N-terminal polyproline peptide from a 60 kDa lamellipodin fragment is embedded within the BChE tetramer. Four identical BChE subunits are colored blue, red, purple and green. Lamellipodin is colored yellow. The polyproline region is protected from proteolysis by 4 BChE helices. The lamellipodin stalk is vulnerable to proteolysis. Figure from [2].

Our model of the C5 variant of BChE is patterned after the model of collagen-tailed acetylcholinesterase proposed by the laboratory of Joel Sussman and Israel Silman in collaboration with Jean Massoulié [2]. Figure 5 depicts four identical subunits of BChE assembled into a tetramer through interaction of their C-terminal tetramerization domains with a polyproline peptide. It has been shown that for human C4 BChE this polyproline peptide derives from lamellipodin [4,5]. In the case of C5, we propose that the polyproline peptide is part of the C-terminal portion of lamellipodin. This fragment of lamellipodin has a mass of ~60 kDa [23]. Our model is consistent with our mass spectral and Western blotting observations, which indicate that the C-terminal portion of lamellipodin is associated with C5 BChE. It is also consistent with earlier genetic analyses that showed both C4 and C5 BChE were under joint control of Chr 3q26.1–q26 (for BChE) and Chr 2q33 (for lamellipodin). Thus, no other protein is required to explain the extra 60 kDa mass found in C5 BChE.

**Figure 6:**
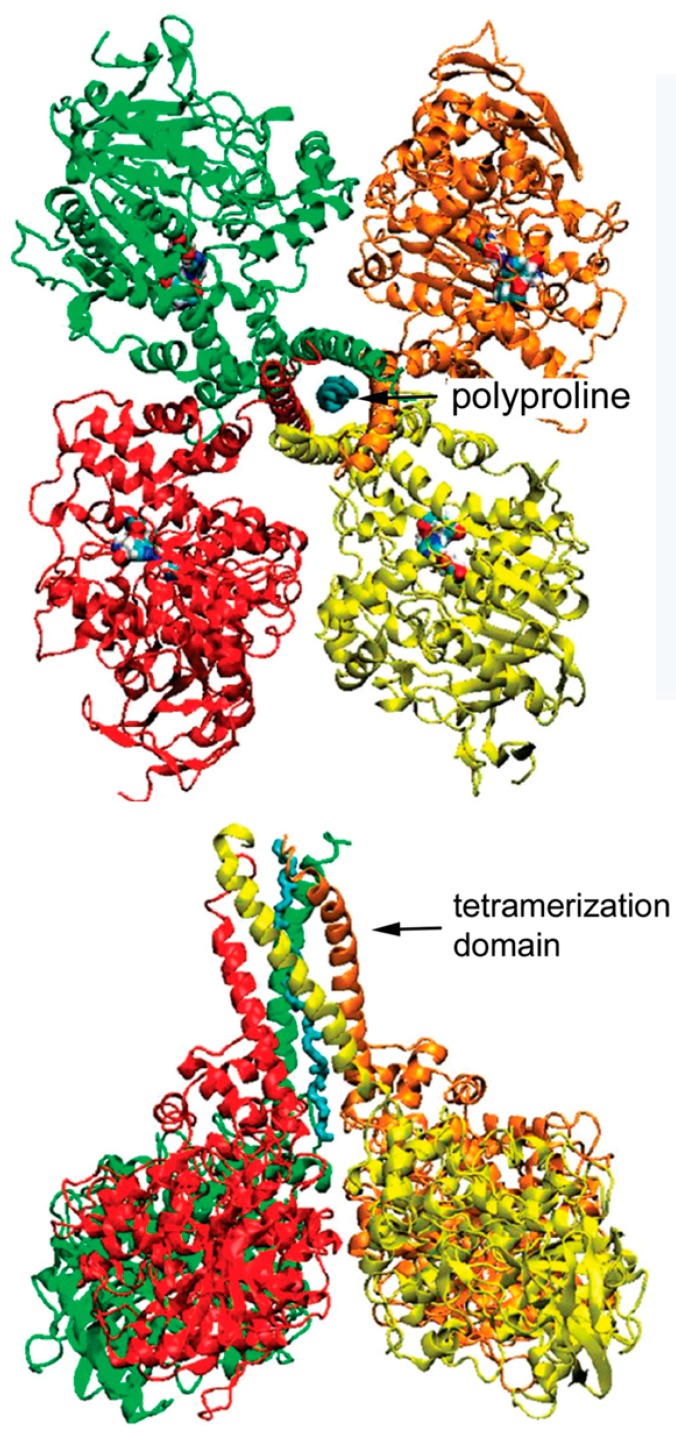
Model of C4 BChE tetramer. The 40-residue tetramerization domain of each BChE subunit folds into a helix. Four helices are held together by interaction with a polyproline-rich peptide. The red, green, brown, and yellow structures represent 4 identical BChE subunits. The blue spiral intertwined with the helices of the tetramerization domain is polyproline. Figure from [29].

The C4 BChE tetramer contains only short polyproline-rich peptides. We propose that the polyproline rich region is protected by helices of the BChE tetramerization domain. The remaining portion of the 60 kDa lamellipodin C-terminus is unprotected and would be vulnerable to proteolysis. Therefore, C5 could be converted to C4 by proteolytic cleavage of the unprotected lamellipodin stalk, leaving only short polyproline-rich peptides in the tetramer (Figure 6).

### 3.2. C5 Expression Is Non-Mendelian

C5 is inherited as an autosomal dominant trait. Studies of the heritability of C5 (also called CHE2) have shown deviations from a Mendelian distribution. In a study of 832 families, 46% of the children had C5 when one parent had C5. Contrary to expectation, when both parents had C5, only 85% of the children had C5 [16]. Family studies of the British population revealed three instances where neither parent of the propositus showed the C5 band [10]. Offspring phenotypes differed significantly from expectation in a study of 451 Brazilian families, there being a dearth of C5 positive children and an excess of C5 negative children [30]. An individual whose blood was phenotyped several times over a 6 month period, had the C5 band disappear and then reappear 2 months later [17]. We propose that the non-Mendelian pattern of inheritance is explained by the instability of the C5 tetramer.

### 3.3. Cord Blood

Nondenaturing gel electrophoresis of umbilical cord plasma showed that 100% of the 103 random samples analyzed had an extra slow moving band of BChE activity. For most individuals, the mobility of that band in fresh cord blood samples was slightly different from the mobility of the C5 band in C5 adult blood. We propose that the extra BChE band in cord plasma is a combination of BChE tetramer and a fragment of lamellipodin, similar to the case for C5 tetramers in adult plasma. The lamellipodin fragment in cord plasma BChE is likely to include additional lamellipodin residues or posttranslational modifications that make it slightly bigger than C5. This slight difference in size between C5 in adult plasma and C5-like BChE in cord plasma suggests that proteases participate in clipping the parent lamellipodin protein. This hypothesis may also explain the existence of other slow-migrating variants such as the Cynthiana variant and other rare variants leading to high BChE activity in blood [31]. The presence or absence of C5 in blood may be controlled by the activity or genetics of one or several proteases.

### 3.4. Motif for Associating Subunits Into Tetramers

**Figure 7:**
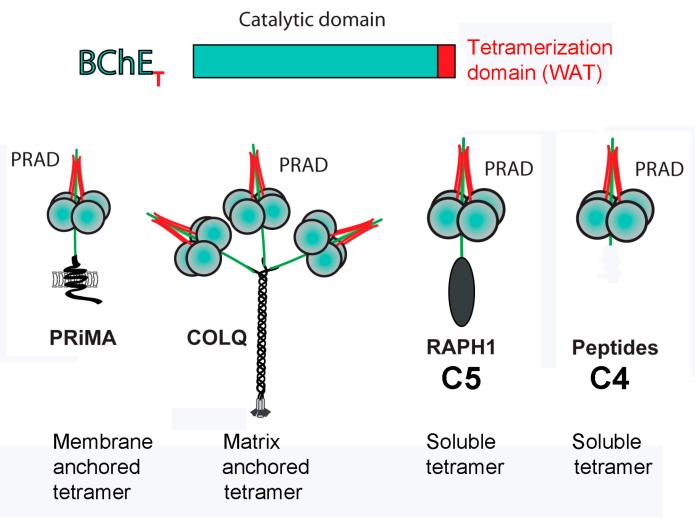
All BChE tetramers are built around proline-rich sequences. The tetramerization domain (WAT) [32] is at the C-terminus of the BChE protein. The proline-rich attachment domain (PRAD) of the proteins PRiMA (Q86XR5), COLQ (Q9Y215), and lamellipodin (RAPH1, Q70E73) organizes the subunits into a tetramer by interaction with the tetramerization domain. AChE makes similar heteromeric tetramers [35], with the exception of C5, which has not yet been found in AChE.

To date BChE and AChE tetramers are the only proteins known to use a C-terminal tetramerization domain in combination with a polyproline-rich peptide to assemble 4 subunits into a tetramer [32,33,34]. The polyproline-rich peptide can be part of a longer protein as in ColQ, PRiMA, and lamellipodin (*RAPH1*), or it can be a short 15 residues, as illustrated in Figure 7. The association is noncovalent but is very tight based on the observation that AChE and BChE tetramers do not dissociate in dilute solution. The crystal structure of the tetramerization domain in complex with a 15-residue polyproline-rich peptide is available [2]. We anticipate that assembly into tetramers through binding of a polyproline-rich peptide will be recognized in the future as a general motif for tetramer formation in other proteins.

### 3.5. Potential Clinical Application

The human BChE tetramer has a long half-life in the circulation of about two weeks [36]. This long half-life is explained as follows: (1) The BChE tetramer has a molecular weight of 340 kDa. Large molecules are resistant to clearance by the kidneys; (2) The 36 N-linked carbohydrate chains on the surface of the tetramer provide a sugar coating that protects the BChE tetramer from proteolysis.

We propose that human BChE can be used as a carrier to deliver proteins or hormones that have a short half-life in circulation. By combining with BChE, the half-life of these compounds in circulation may be increased. If 5 to 17 proline residues are added to a therapeutic protein and this construct is mixed with recombinant BChE monomers, the therapeutic protein should become embedded in the BChE tetramer. The BChE complex may last days rather than minutes in circulation.

## 4. Materials and Methods

### 4.1. Human Plasma

Five 330 mL bags of volunteer plasma from adult donors in citrate phosphate dextrose anticoagulant were from the University of Nebraska Medical Center Blood Bank. This plasma was shown to contain the C5 form of BChE by native PAGE stained for BChE activity (see below for details). The once-frozen plasma had been thawed by the Blood Bank before we received it. Umbilical cord blood in EDTA anticoagulant from 103 individuals was never frozen. The blood bank donated the cord plasma after it had been stored at 4 °C for 2–4 weeks. A second set of cord EDTA plasma was stored at −20 °C for 5 years. Blood from one adult C5 donor was collected into EDTA anticoagulant. It was used fresh and up to 2 years while stored at 4 °C or at −20 °C for 9 years. Research on unidentified human samples is exempt from Code of Federal Regulations 45CFR46§46.101.

### 4.2. C5 Phenotyping

The C5 band was visualized on 4–30% polyacrylamide gradient gels, topped with a 4% stacking gel. Gels were prepared in an SE600 Hoefer gel apparatus. Wells were loaded with 3 µL plasma diluted to 20 µL with bromphenol blue and glycerol. Following electrophoresis at 4 °C for 20 h at 250 volts constant voltage, gels were stained for BChE activity by the method of Karnovsky and Roots [37] with butyrylthiocholine iodide as the substrate.

### 4.3. BChE Activity

BChE activity was measured in 0.1 M potassium phosphate pH 7.0 at 25 °C with 1 mM butyrylthiocholine iodide in the presence of 0.5 mM 5,5′-dithiobis (2-nitrobenzoic acid) on a Gilford spectrophotometer interfaced to a MacLab data recorder (ADinstruments, Inc., Colorado Springs, CO, USA). The increase in absorbance at 412 nm was converted to micromoles butyrylthiocholine hydrolyzed using the extinction coefficient 13,600 M^−1^ cm^−1^ [38]. Units of activity are expressed as micromoles per min. BChE units/mL were converted to mg/mL using the conversion factor of 500 units/mg.

### 4.4. BChE Purified from Cohn Fraction IV-4

Human plasma-derived BChE was purified in a 2-step protocol, similar to that of [39]. In brief, 80 kg of Cohn paste were extracted with water, filtered, chromatographed on Q-ceramic ion exchange medium at pH 4.5 and on a hupresin affinity column at pH 8.

### 4.5. Purification of C5 from Plasma on Hupresin-Sepharose

Hupresin was synthesized by Emilie David, Chemforase, Mont-Saint-Aignan, France emilie.david@chemforase.com. A 0.25 mL slurry of hupresin–Sepharose containing 0.2 mL settled gel in PBS was incubated with 1 mL of C5 EDTA plasma, or 1 mL of cord EDTA plasma, or 1 mL of adult EDTA plasma. Samples were rotated at room temperature for 1 h. The beads were transferred to a Millipore^®^ PVDF 0.45 µm Ultrafree^®^-MC-HV centrifugal filter (EMD Millipore, Billerica, MA, USA, UFC30HV00) and washed with 0.5 mL aliquots of PBS twelve times until absorbance at 280 nm of the flow through was near zero. BChE was released from the washed hupresin beads into fresh microfuge tubes with 0.2 mL of 0.1 M procaine in 0.1 M TrisCl pH 7.5. The first extraction yielded 80% and a second extraction yielded 20% of the bound BChE activity. Both C5 and C4 BChE isozymes were present in the procaine eluent. This affinity purification step removed 90% of the plasma proteins, leaving plasminogen as the major contaminant.

### 4.6. Immunopurification of BChE

Mouse anti-human BChE monoclonal antibody B2 18–5 (accession KT189143 heavy chain, KT189144 light chain) was characterized in-house [40]. The nucleotide and amino acid sequences are posted on the National Center for Biotechnology Information website. The antibody was immobilized on CNBr-activated Sepharose 4B and stored in phosphate buffered saline plus 0.1% azide. A 0.2 mL suspension contained 100 µg antibody immobilized on 0.04 mL Sepharose beads. Hupresin-purified BChE containing C4 and C5 isozymes was diluted 5-fold with water to reduce the procaine concentration to 0.02 M. A 1 mL solution of BChE in 0.02 M procaine, 0.02 M TrisCl pH 7.5 was rotated with 0.04 mL of B2 18–5 Sepharose beads at room temperature for 4 h. Activity assay of the unbound supernatant showed that 97% of the BChE was bound to the antibody beads. The antibody beads were transferred to a Millipore^®^ PVDF 0.45 µm Ultrafree^®^-MC-HV centrifugal filter, washed with PBS five times to remove procaine, and washed with water twice to remove salts. BChE, including C5, was released from the antibody beads with 100 µL of 50% acetonitrile, 1% TFA into new tubes. The extract was dried and prepared for trypsin digestion.

### 4.7. Trypsin Digestion

The dried extracts were dissolved in 25 µL of 20 mM ammonium hydrogen carbonate pH 8.1 and digested with 1 µL (0.4 µg) porcine trypsin (Promega, Madison, WI, USA, V511C) overnight. The digest solution was centrifuged at 14,000 rpm for 30 min to pellet particulates before 10 µL was transferred to autosampler vials for mass spectral analysis.

### 4.8. Liquid Chromatography-Tandem Mass Spectrometry (LC-MS/MS)

Electrospray ionization mass spectrometry was performed on a 6600 Triple TOF mass spectrometer (Sciex, Framingham, MA, USA). Five microliters of sample were subjected to HPLC separation using a cHiPLC Nanoflex microchip column (Eksigent, Dublin CA, USA) attached to a splitless Ultra 1D Plus ultra-high pressure chromatography system (Eksigent, Dublin, CA, USA). The Nanoflex microchip system consisted of a replaceable microfluidic trap column (200 µm × 0.5 mm) and separation column (75 µm × 15 cm separation) both packed with ChromXP C18 resin (3 µm, 120 Å particles). The sample was loaded onto the trap column and washed for 15 min at 2 µL/min with 0.1% formic acid in water to remove salts. The flow rate was reduced 0.3 µL/min, and the sample was eluted using a 65 min gradient ranging from 95% solvent A (0.1% formic acid in water) plus 5% solvent B (0.1% formic acid in acetonitrile) to 70% solvent A plus 30% solvent B. Effluent from the HPLC column was sprayed directly into the mass spectrometer. Mass spectra were collected in positive mode, over a mass range of 200 to 2000 Da, using an accumulation time of 250 ms, a collision energy of 10 volts, a declustering potential of 60 volts, an ion spray potential of 2700 volts, and an interface heater temperature of 150 °C. Peptide fragmentation was accomplished by collision-induced dissociation using nitrogen as the collision gas at a pressure of 2 × 10^−5^ Torr. Fragmentation spectra were collected in positive mode, over a mass range of 50–2000 Da, using an accumulation time of 25 ms, a collision energy determined by the software (rolling), and a collision energy spread of ±15 volts. Peptides to be fragmented were chosen by an information directed acquisition algorithm using charge state 1 to 4 and minimum signal 100 cps. Up to 50 fragmentation spectra were collected in each cycle with target ions being excluded for 5 s after the second acquisition. Masses within 6 Da of a target ion were excluded. The Paragon algorithm in Protein Pilot software 4.0.8085 (AB Sciex, Framingham, MA, USA) was used to identify the proteins and to assign confidence levels. Database search parameters included Sample type = identification, Cys alkylation = none, Digestion = none, Instrument = Triple TOF 6600, Special factors = none, Species = Homo sapiens, ID focus = biological modifications, Database = uniprot_sprotJAN2015.fasta, Search effort = thorough, and FDR analysis = active.

BChE from fresh, never-frozen C5 and cord plasma samples was analyzed by mass spectrometry. C5 from 3 adult donors was analyzed 10 times. Cord BChE was analyzed 6 times. The cord BChE was prepared from 5 individual umbilical cord plasma samples represented by lanes 1–3 in Figure 2, and from a pool of 6 cord plasma samples. 

### 4.9. Capillary Electrophoresis Simple Western Blot

Human BChE was purified from 1 mL each of C5 EDTA plasma, cord EDTA plasma, and wild-type EDTA plasma on hupresin eluted with 0.1 M procaine in 0.1 M TrisCl pH 7.5. About 2 units of BChE activity representing 4 µg BChE protein were dialyzed and concentrated to 100 µL in a Microcon YM-30 centrifugal filter (EMD Millipore, 42410, regenerated cellulose, 30,000 MWCO). The samples were boiled for 3 min to denature the proteins because gel shift studies had shown that the RAPH1 antibody does not recognize native C5. The anti-RAPH1 polyclonal antibody in rabbit (Sigma, St Louis, MO, USA; HPA020027) was made against amino acids 1105–1248 at the C-terminus of human lamellipodin [41]. The Simple Western Testing Service personnel at RayBiotech, Inc. (Norcross, GA, USA) loaded 40 nL of sample and antibody into an assay plate in the WES machine (ProteinSimple, San Jose, CA, USA). Proteins were separated by size through a stacking and separation matrix in the capillary, and immobilized to the capillary wall via a proprietary, photoactivated capture chemistry. Proteins that bound the anti-RAPH1 polyclonal antibody (diluted 1:3000) were immunoprobed using an HRP-conjugated secondary antibody and chemiluminescent substrate.

### 4.10. Genotyping Analysis

Blood samples were collected from two C5 adults, who signed informed consent to participate in the genetic study. Genomic DNA was extracted from peripheral blood leucocytes with the QIAamp Blood-DNA mini reagent kit (Qiagen, Courtaboeuf Cedex, France) according to the manufacturer’s instructions. DNA was eluted in 100 µL of water in the final step and stored at −20 °C until required. Sequencing of the coding exons and exon-intron boundaries of the *BCHE* (NM_000055.3), *RNPEP* (NM_001319183.1), and *RAPH1* (NM_203365.3) genes was performed after PCR amplification of genomic DNA, with specific primers (list on request). PCR products were purified with use of the High Pure PCR Product Purification Kit (Roche Diagnostics, Mannheim, Germany) according to the manufacturer’s instructions. Both strands were sequenced using amplification primers as sequencing primers and BigDyeDeoxy terminator cycle sequencing according to the manufacturer’s instruction (Life Technologies, Aubin, France). Purified sequencing fragments were separated by capillary electrophoresis and detected by laser-induced fluorescence on a 3500 Dx Genetic Analyzer (Thermo Fisher Scientific, Villebon-sur-Yvette, France).
